# Supplementary material for: Multi-color dual wavelength vat photopolymerization 3D printing via spatially controlled acidity
Source: Nat Commun. 2024 May 8;15:3867. doi: 10.1038/s41467-024-48159-7 (PMC11078982; doi:10.1038/s41467-024-48159-7)
Supplement: Supplementary file 1 — Supplementary information [file 41467_2024_48159_MOESM1_ESM.pdf]

Supplementary Information

Multi-Color Dual Wavelength Vat Photopolymerization 3D Printing Via Spatially Controlled Acidity

Kyle C. H. Chin[1], Grant Ovsepyan[1], Andrew J. Boydston[1,2,3*]

[1]*Department of Chemical and Biological Engineering, University of Wisconsin, Madison, WI, 53706, USA*

[2]*Department of Chemistry, University of Wisconsin, Madison, WI, 53706, USA*

[3]*Department of Materials Science and Engineering, University of Wisconsin, Madison, WI, 53706, USA*

**Corresponding authors, email: aboydston@wisc.edu*

Table of Contents

Supplementary Fig. 1: Chemical structures and acronyms 3

Supplementary Fig. 2: TAS Solubility Experiment in Various Monomers 4

Supplementary Table 1: Print parameters for resins printed with Elegoo Mars 3 printer 4

Supplementary Fig. 3: Additional F1 printed objects 5

Supplementary Fig. 4: Additional resolution images 6

Supplementary Fig. 5: Light penetration color change images 7

Supplementary Fig. 6: Light penetration color change MATLAB 8

Supplementary Fig. 7: Wavelength dependent color change 9

Supplementary Fig. 8: UV-Vis spectroscopy of TAS, avobenzone 10

Supplementary Fig. 9: Samples printed from F1 after being stored either in dark or in ambient light 11

Supplementary Fig. 10: Color tracking of 3D printed samples from F1 after being stored either in dark or in ambient light 12

Supplementary Fig. 11: Sample stability with avobenzone compared to without 13

Supplementary Fig. 12: Images of submerged parts 14

Supplementary Fig. 13: UV-Vis spectra of water with parts submerged 14

Supplementary Fig. 14: Resolution test prints of F3 and F4 15

Supplementary Fig. 15: Color change of 1:3 and 3:1 ratio BG and MR F4 samples 16

Supplementary Fig. 16: Flowchart for photopatterning 17

Supplementary Fig. 17: Custom dual-wavelength 3D printer 18

Supplementary Fig. 18: Custom Printer Projected Image Alignment 19

Supplementary Table 2: Printing parameters for dual wavelength printer 21

Supplementary Fig. 19: Tensile data for multicolor samples 22

Supplementary Fig. 20: Additional dual-wavelength 3D printed objects 23

Supplementary Fig. 21: White Lamp Light Source Emission Spectra 24

Supplementary Fig. 22: 365 nm Kessil Lamp Light Source Emission Spectra 24

Supplementary Fig. 23: 456 nm Kessil Lamp Light Source Emission Spectra 25

Supplementary Fig. 24: 405 nm Kessil Lamp Light Source Emission Spectra 25

Supplementary Fig. 25: 365 nm Kessil Lamp Light Source Emission Spectra 26

Supplementary Fig. 26: White Light Projector Emission Spectra 26

Supplementary Fig. 27: Elegoo Mars 3 LCD 3D Printer Emission Spectra 27

Design Files 27

Supplementary Fig. 1: Chemical structures and acronyms

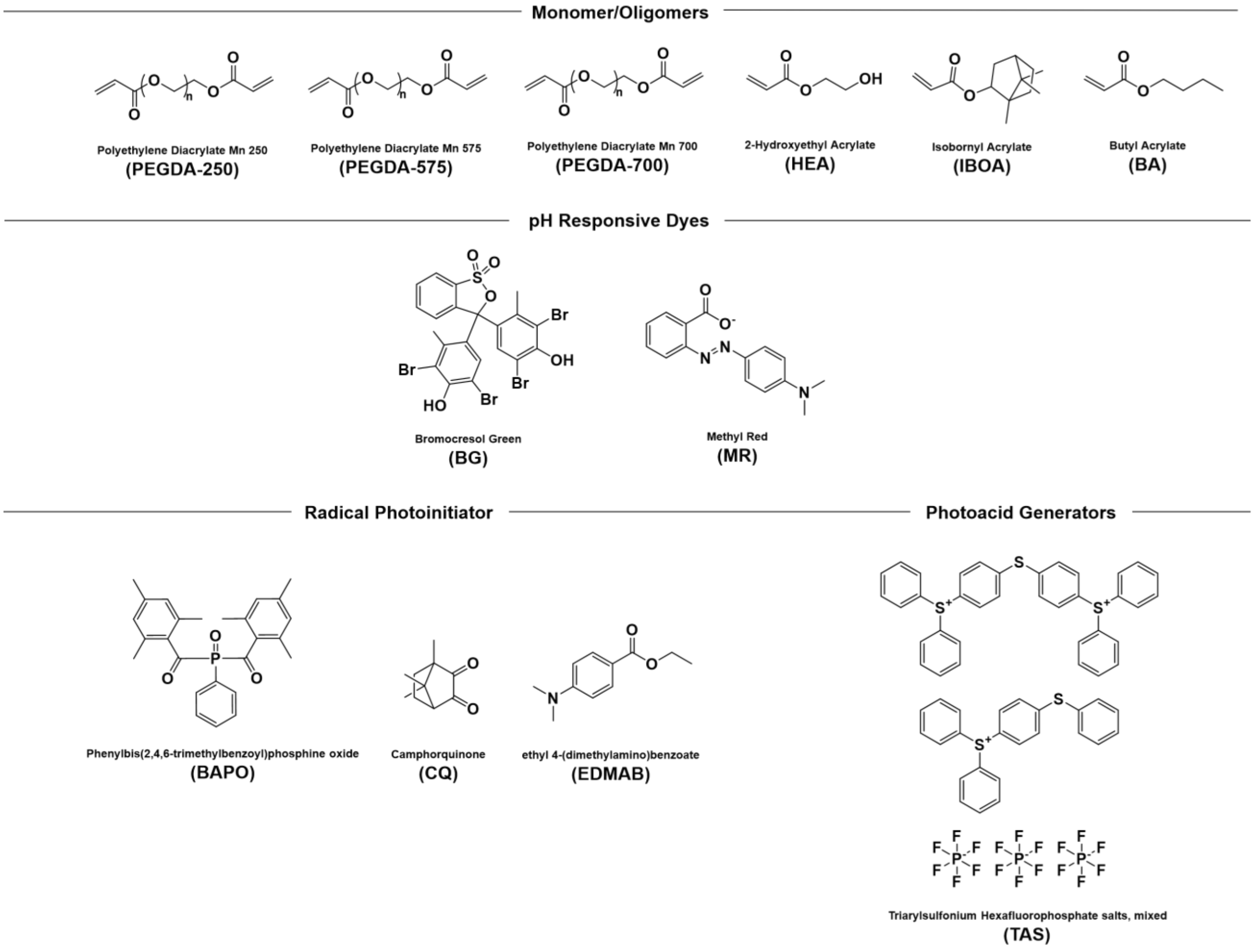

Supplementary Fig. 1: Compounds included in resin formulations along with their structures, names, and abbreviations.

Supplementary Fig. 2: TAS Solubility Experiment in Various Monomers

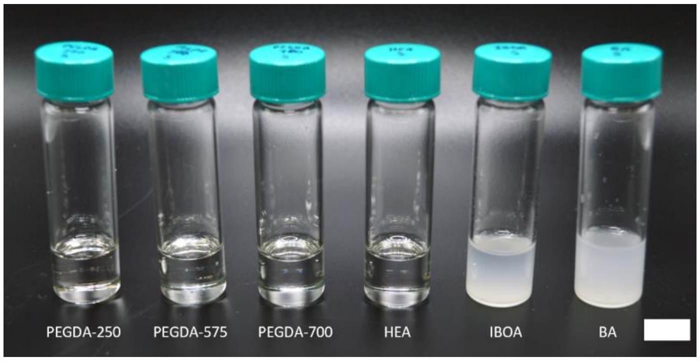

Supplementary Fig. 2: Solubility of TAS in PEGDA-250, PEGDA-575, PEGDA-700, HEA, IBOA, and BA at a concentration of 3 wt% compared to monomer. Scale bar= 10 mm

Supplementary Table 1: Print parameters for resins printed with Elegoo Mars 3 printer

Supplementary Table 1: Print parameters for each resin printed on a Elegoo Mars 3 printer.

Resin	Normal Layer Time (sec)	Bottom Layer Time (sec)	# Bottom Layers	Layer Thickness (µm)	Lift Distance (mm)	Lift Speed (mm/sec)	Retract Speed (mm/sec)
F1	12	30	6	50	6	60	150
F2	--	--	--	--	--	--	--
F3	12	30	6	50	6	60	150
F4	12	120	8	50	6	60	150
F5	--	--	--	--	--	--	--

Supplementary Fig. 3: Additional F1 printed objects

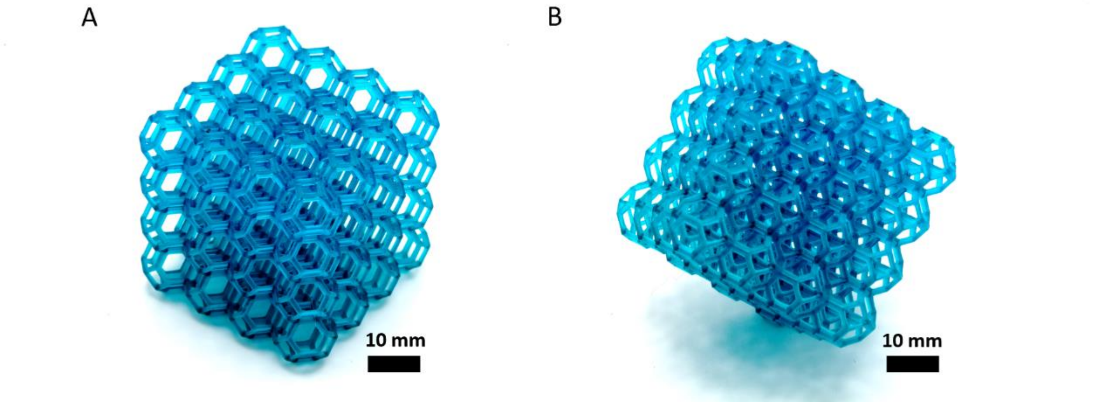

Supplementary Fig. 3: Additional images of complex lattice printed from F1 resin. A) Image taken from above a lattice 3D printed from F1 resin. B) Image taken from the side of a lattice 3D printed from F1 resin.

Supplementary Fig. 4: Additional resolution images

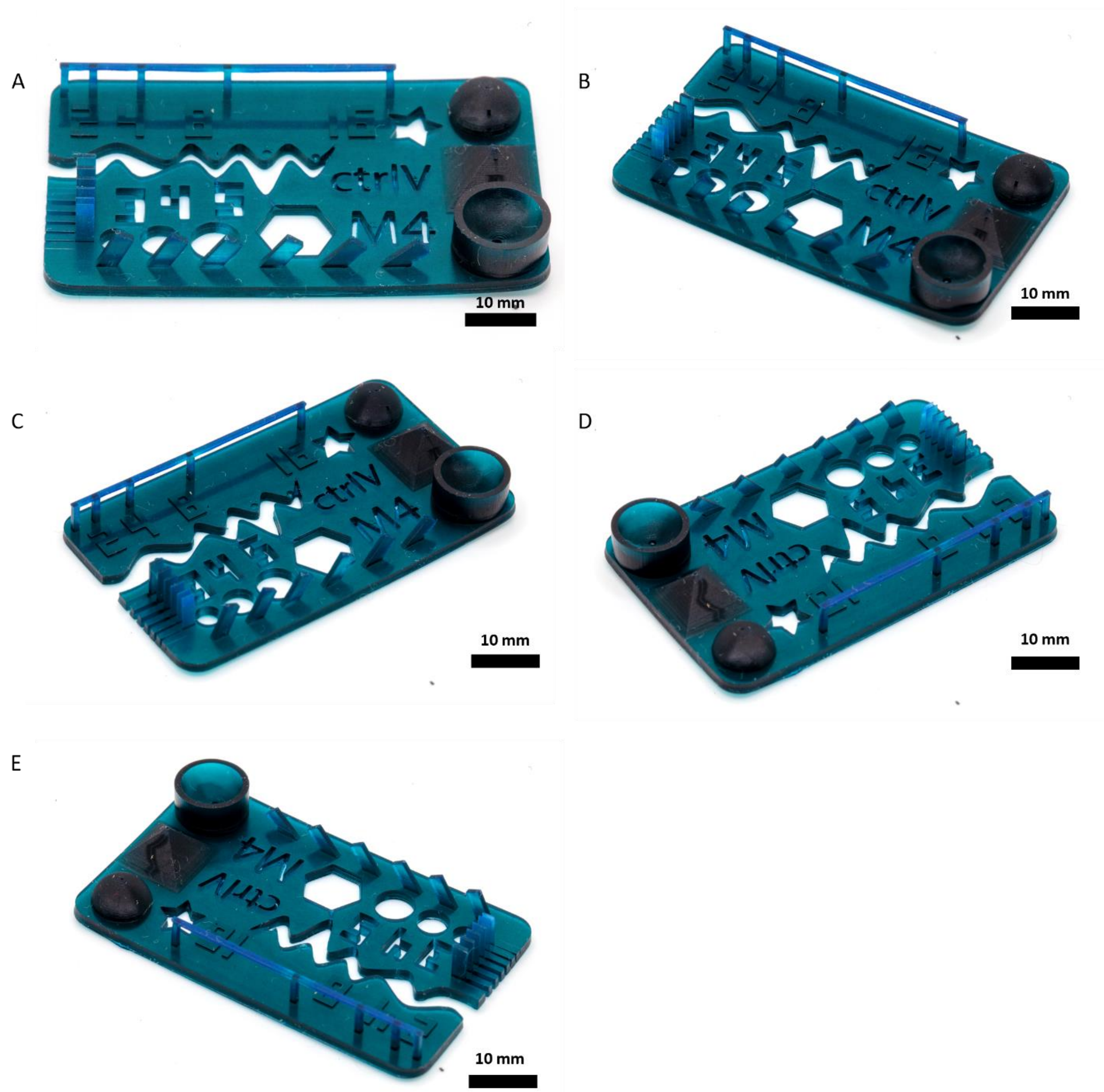

Supplementary Fig. 4: Image of a resolution test object 3D printed from F1 resin taken from different angles. A) front B) tilted right C) tilted left D) back tiled left E) back titled right

Supplementary Fig. 5: Light penetration color change images

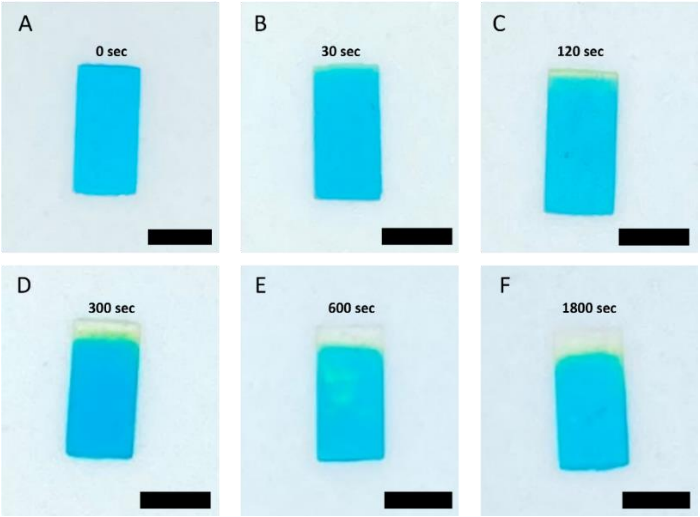

Supplementary Fig. 5: Images of the resulting samples for determining depth of color change with increasing exposures of light. Scale bar= 10 mm A) 0 sec B) 30 sec C) 120 sec D) 300 sec E) 600 sec F) 1800 sec

Supplementary Fig. 6: Light penetration color change MATLAB

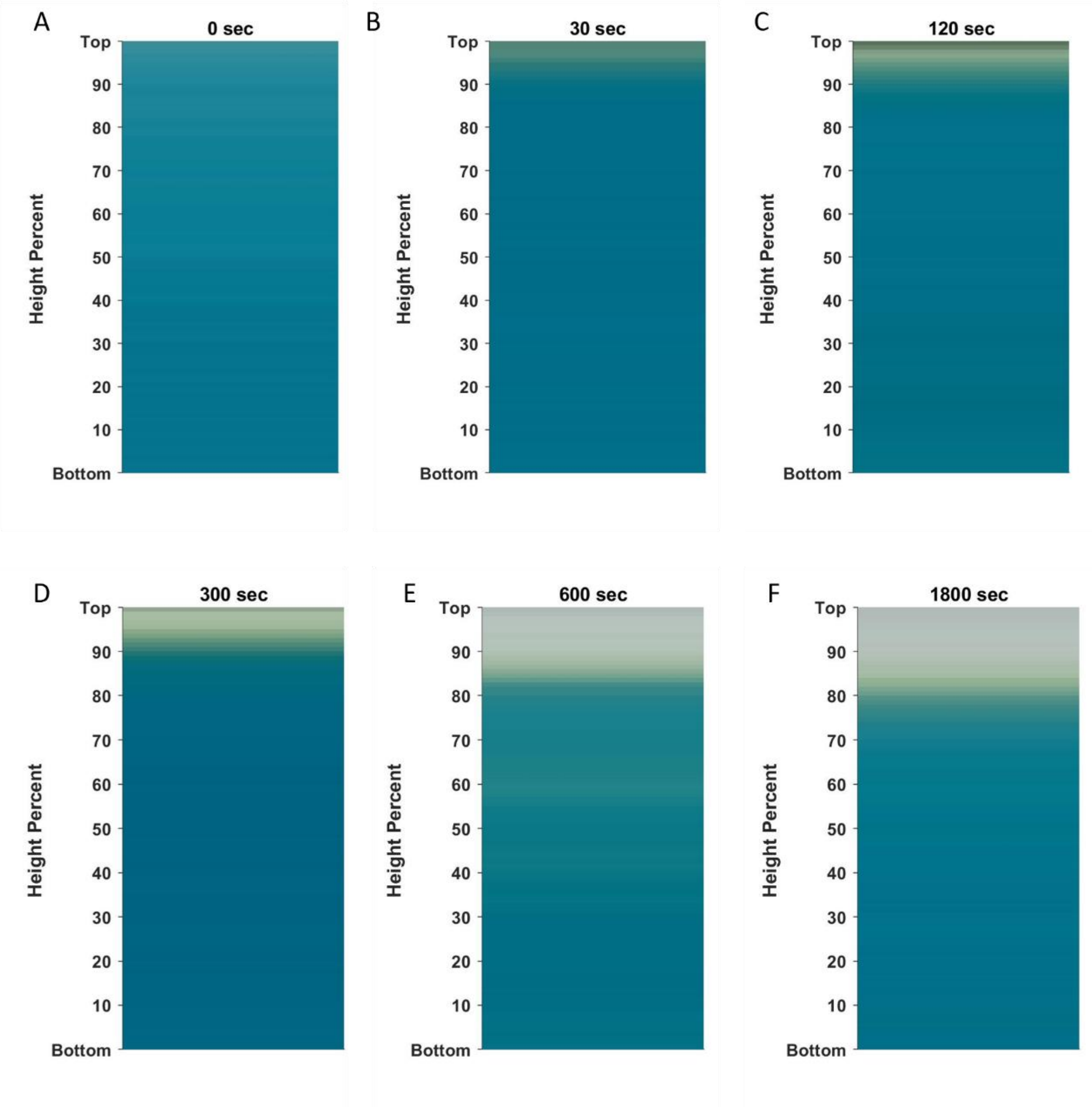

Supplementary Fig. 6: MATLAB extracted images for samples in figure S4 for determining depth of color change with increasing exposures of light. A) 0 sec B) 30 sec C) 120 sec D) 300 sec E) 600 sec F) 1800 sec

Supplementary Fig. 7: Wavelength dependent color change

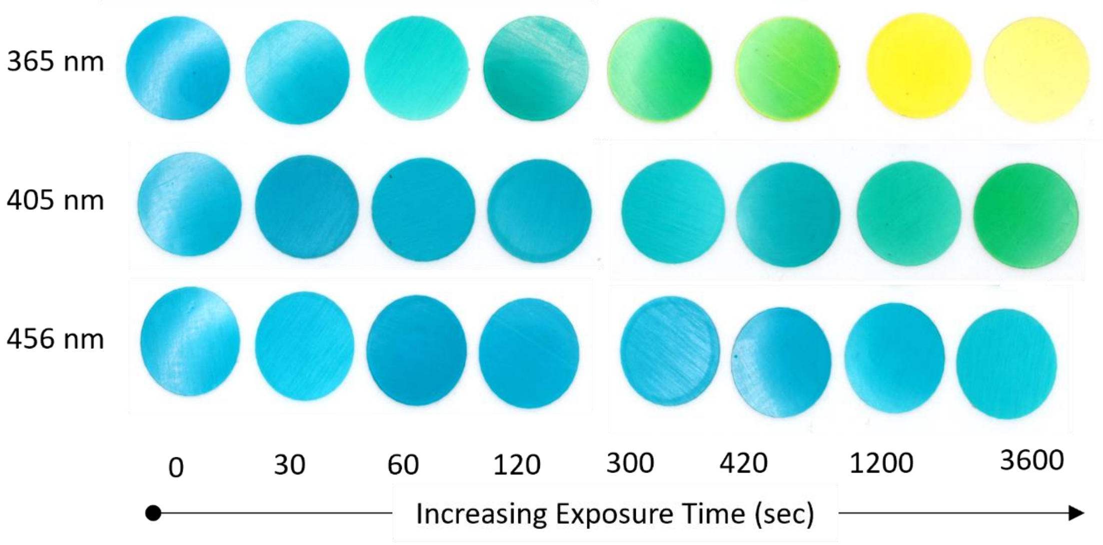

Supplementary Fig. 7: Image of 3D printed samples exposed to various exposures of 10 mW/cm^2 light at either 365 nm (top) 405 nm (middle) or 456 nm (bottom) light.

Supplementary Fig. 8: UV-Vis spectroscopy of TAS, avobenzone

A

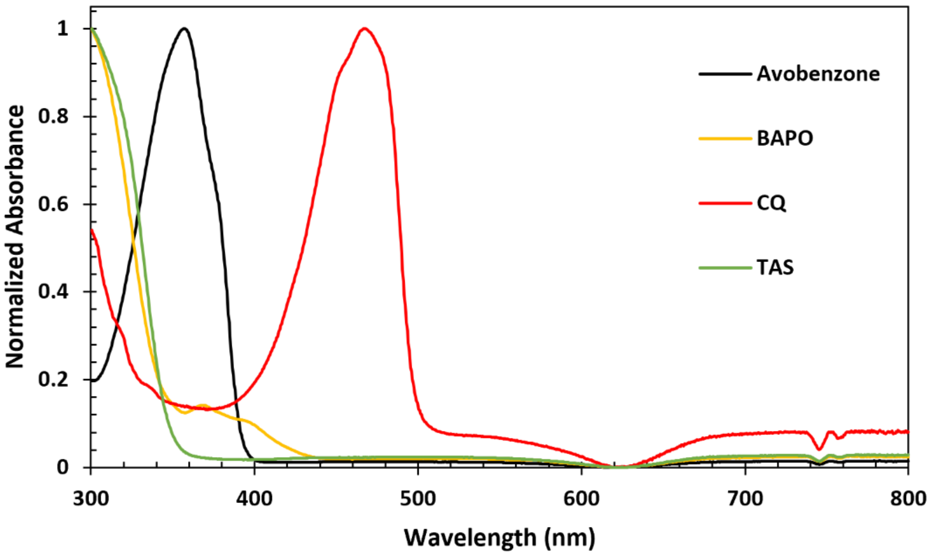

B

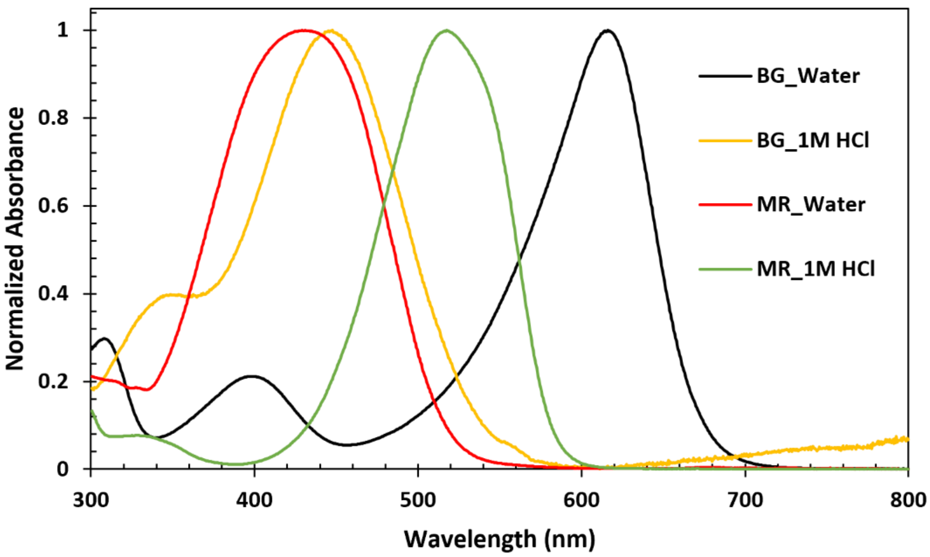

Supplementary Fig. 8: UV-Vis spectroscopy results showing the normalized absorbance v wavelength for A) Avobenzone, BAPO, CQ, and TAS with acetonitrile as solvent B) BG in deionized water, BG in an aqueous 1M HCl solution, MR in deionized water, and MR in an aqueous 1M HCl solution.

Supplementary Fig. 9: Samples printed from F1 after being stored either in dark or in ambient light

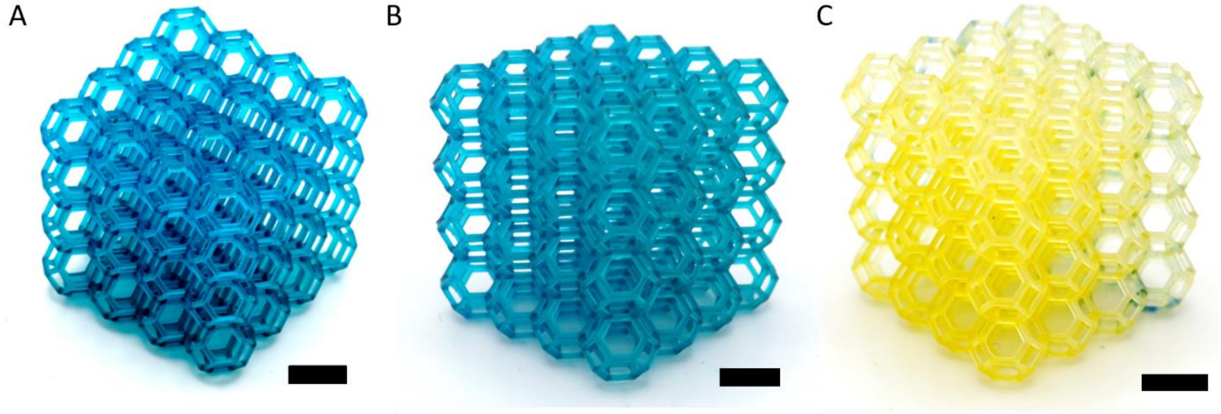

Supplementary Fig. 9: 3D printed lattice from F1 resin. A) As printed B) After 6 weeks stored in the dark C) After 6 weeks stored under ambient lighting

Supplementary Fig. 10: Color tracking of 3D printed samples from F1 after being stored either in dark or in ambient light

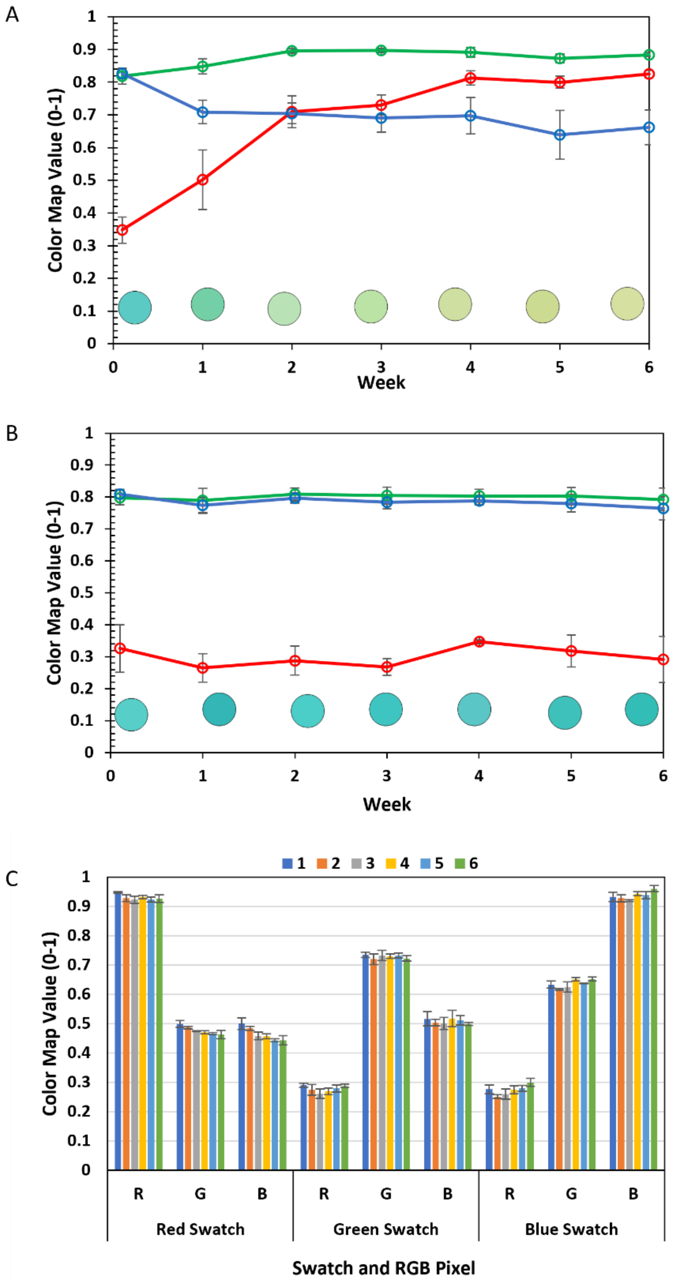

Supplementary Fig. 10: A) RGB value tracking of color change of 3D printed samples from F1 resin over the course of six weeks stored under ambient lighting. Colored circles show the MATLAB extracted value for the color of each sample taken from 10 averaged pixel values across three separate samples. Error bars represent standard deviation form these three replicates. B) RGB value tracking of color change of 3D printed samples from F1 resin over the course of six weeks stored in the dark. Colored circles show the MATLAB extracted value for the color of each sample taken from 10 averaged pixel values across three separate samples. Error bars represent standard deviation form these three replicates. C) RGB values for the color swatches used to over the course of six weeks.

Supplementary Fig. 11: Sample stability with avobenzone compared to without

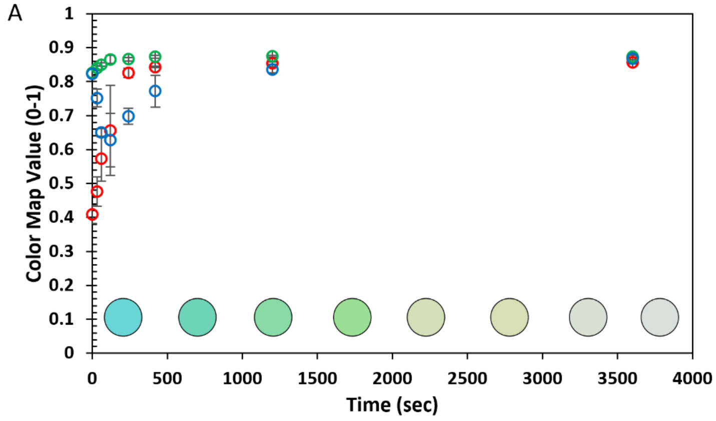

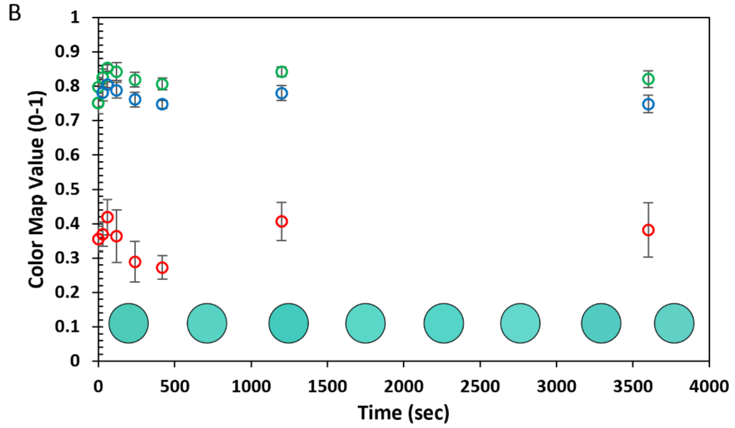

Supplementary Fig. 11: Color stability of samples printed with and without 0.2 wt% avobenzone. A) RGB value tracking of color change of 3D printed samples from F1 resin with increasing exposure to 10 mW/cm2 365 nm light. Colored circles show the MATLAB extracted value for the color of each sample taken from 10 averaged pixel values across three separate samples. B) RGB value tracking of color change of 3D printed samples from F1 resin with 0.2 wt% avobenzone added with increasing exposure to 10 mW/cm2 365 nm light. Colored circles show the MATLAB extracted value for the color of each sample taken from 10 averaged pixel values across three separate samples.

Supplementary Fig. 12: Images of submerged parts

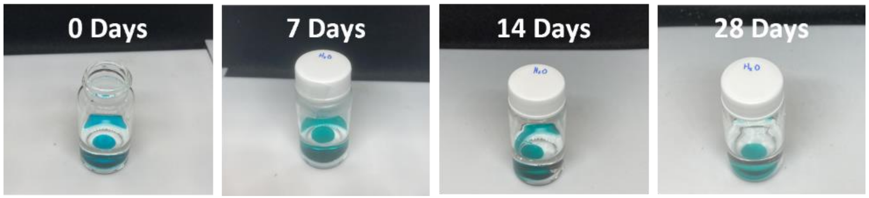

Supplementary Fig. 12: Images of F1 printed samples submerged into water for 0, 7, 14, and 28 days.

Supplementary Fig. 13: UV-Vis spectra of water with parts submerged

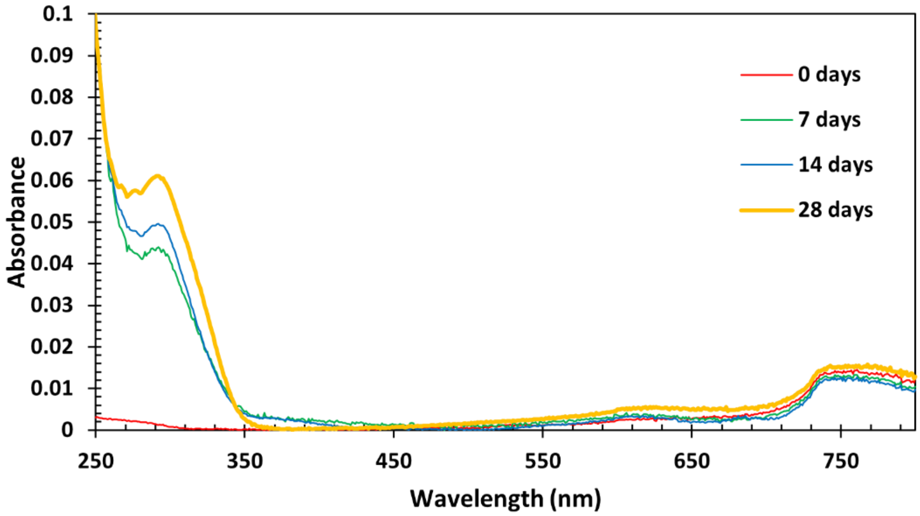

Supplementary Fig. 13: UV-Vis spectra of solution with printed F1 samples submerged at 0, 7, 14, and 28 days. A small peak begins to appear between 550 and 650 nm indicating almost negligible leaching of BG dye into the water.

Supplementary Fig. 14: Resolution test prints of F3 and F4

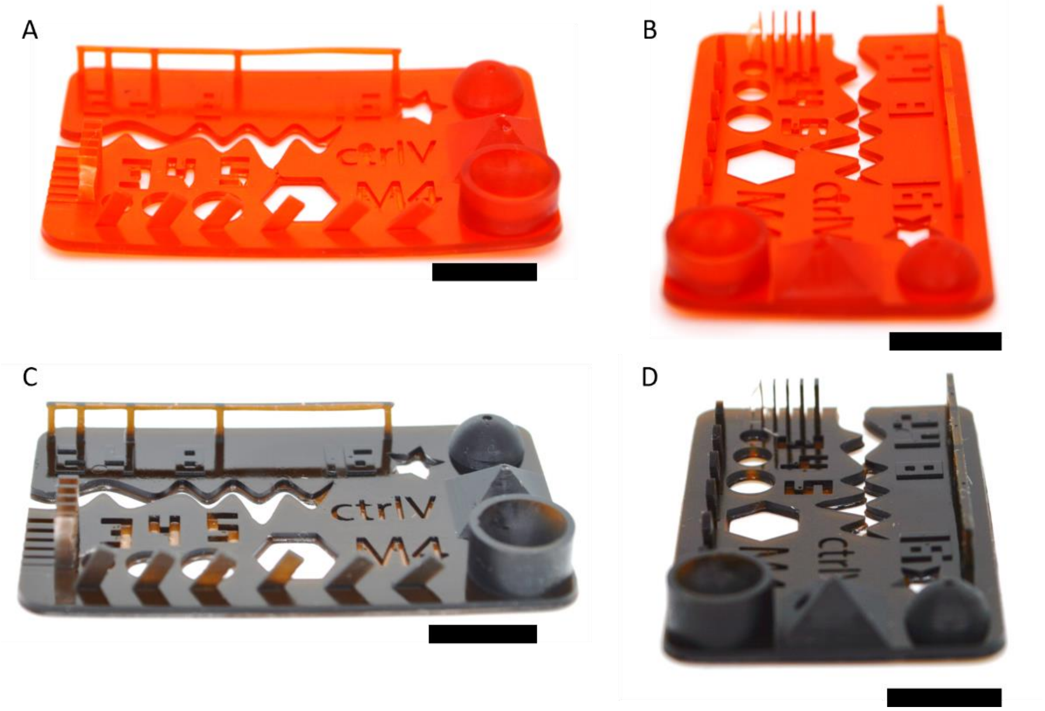

Supplementary Fig. 14: Resolution test prints of F3 and F4 resin printed with a commercial 3D printer. A) F3 front view of test print. B) F3 side view of test print. C) F4 front view of test print. D) F4 side view of test print. Scale bars=10 mm

Supplementary Fig. 15: Color change of 1:3 and 3:1 ratio BG and MR F4 samples

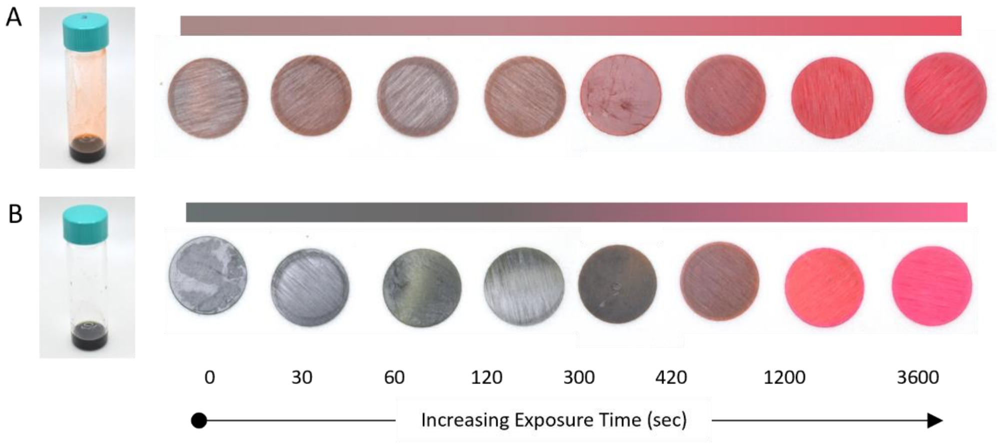

Supplementary Fig. 15: A) Resin formulation for F4 made with a 1:3 ratio of BG and MR. To the right of the resin are printed disks exposed to increasing dosages of UV 365-nm light. B) Resin formulation for F4 made with a 3:1 ratio of BG and MR. To the right of the resin are printed disks exposed to increasing dosages of UV 365-nm light.

Supplementary Fig. 16: Flowchart for photopatterning

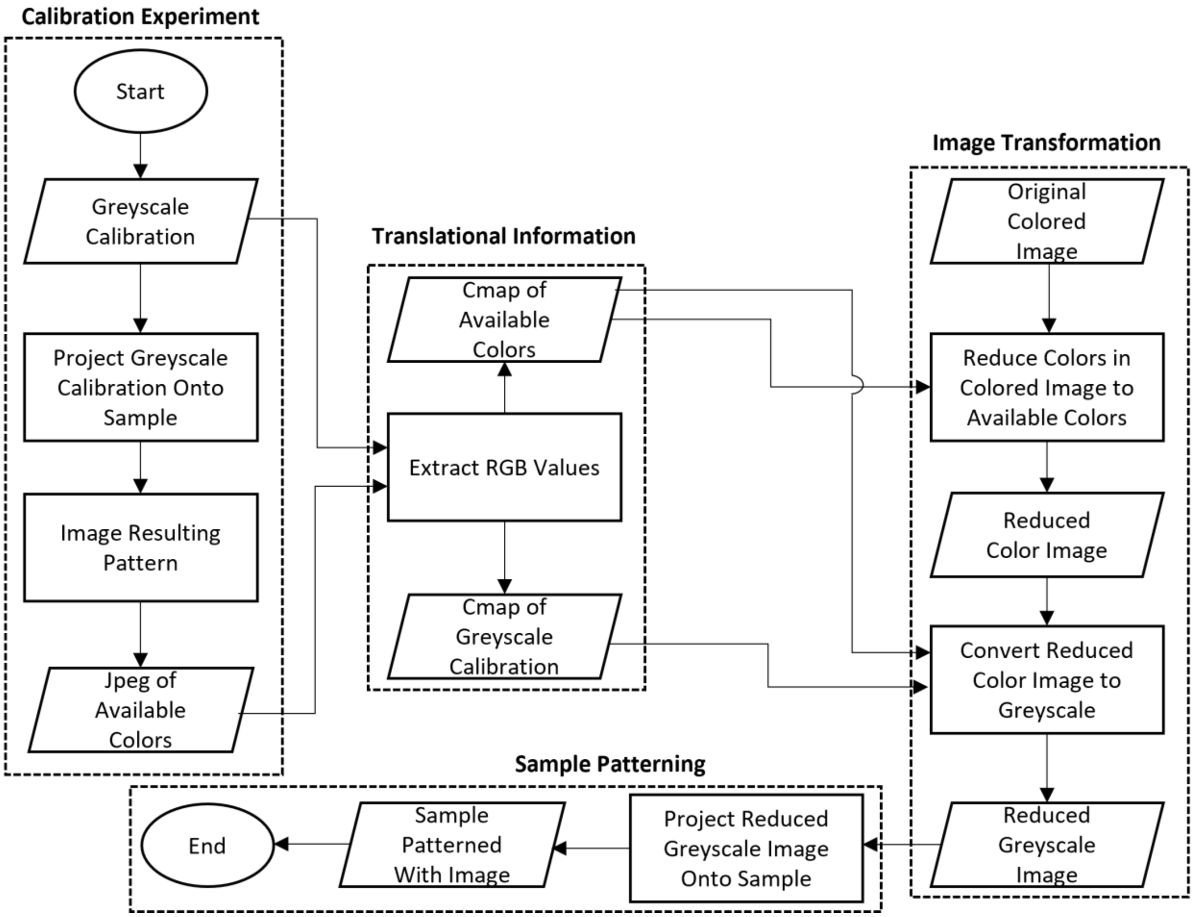

Supplementary Fig. 16: Flowchart of the workflow for photopatterning object printed from multicolor resins using MATLAB.

Supplementary Fig. 17: Custom dual-wavelength 3D printer

A

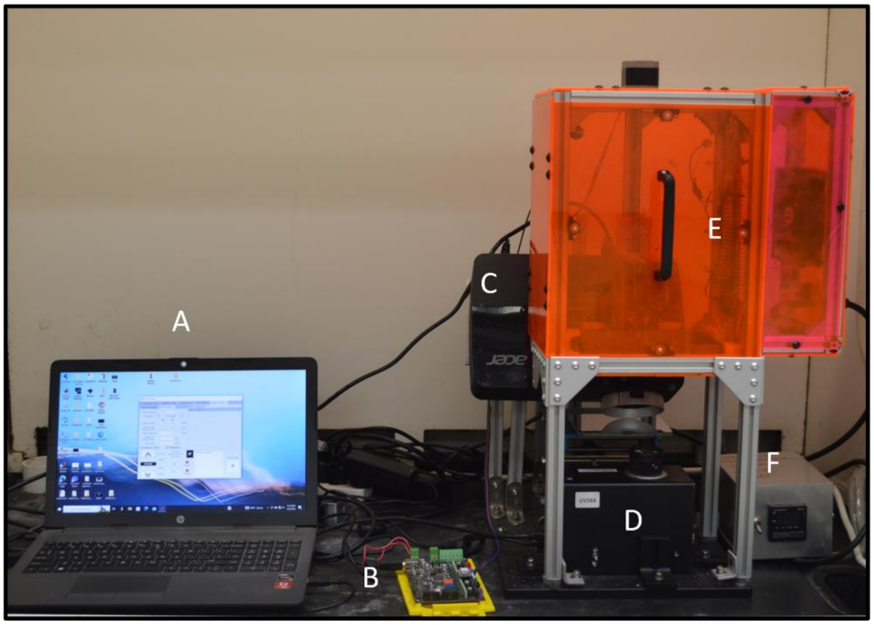

A. Computer displaying MATLAB app for printer operation

B. Controller board

C. White light projector

D. UV light projector with 365 nm LED

E. Printer body

F. Temperature controller

B

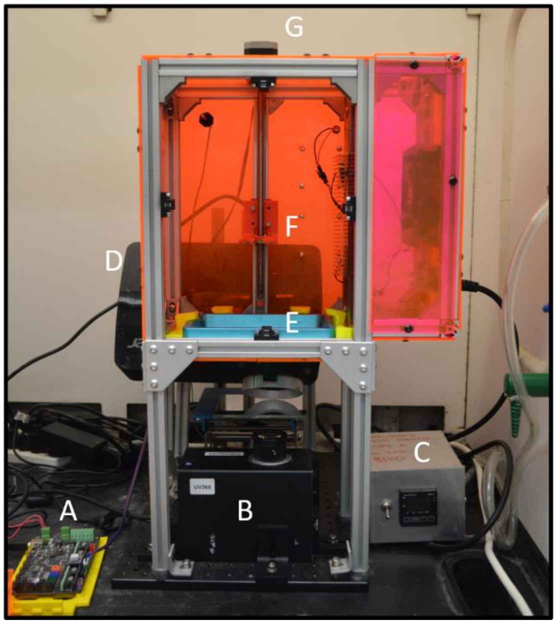

A. Controller board

B. UV light projector with 365 nm LED

C. Temperature controller

D. White light projector

E. Resin vat

F. Build plate attachment point

G. Z-axis stepper motor

Supplementary Fig. 17: Images of the custom dual-wavelength printer with key components labeled. A) Entire 3D printer including the computer with MATLAB app used to operate printer. Key components are labeled to the right of the image. B) Zoomed in image of printer hardware with key components labeled to the right of the image.

Supplementary Fig. 18: Custom Printer Projected Image Alignment

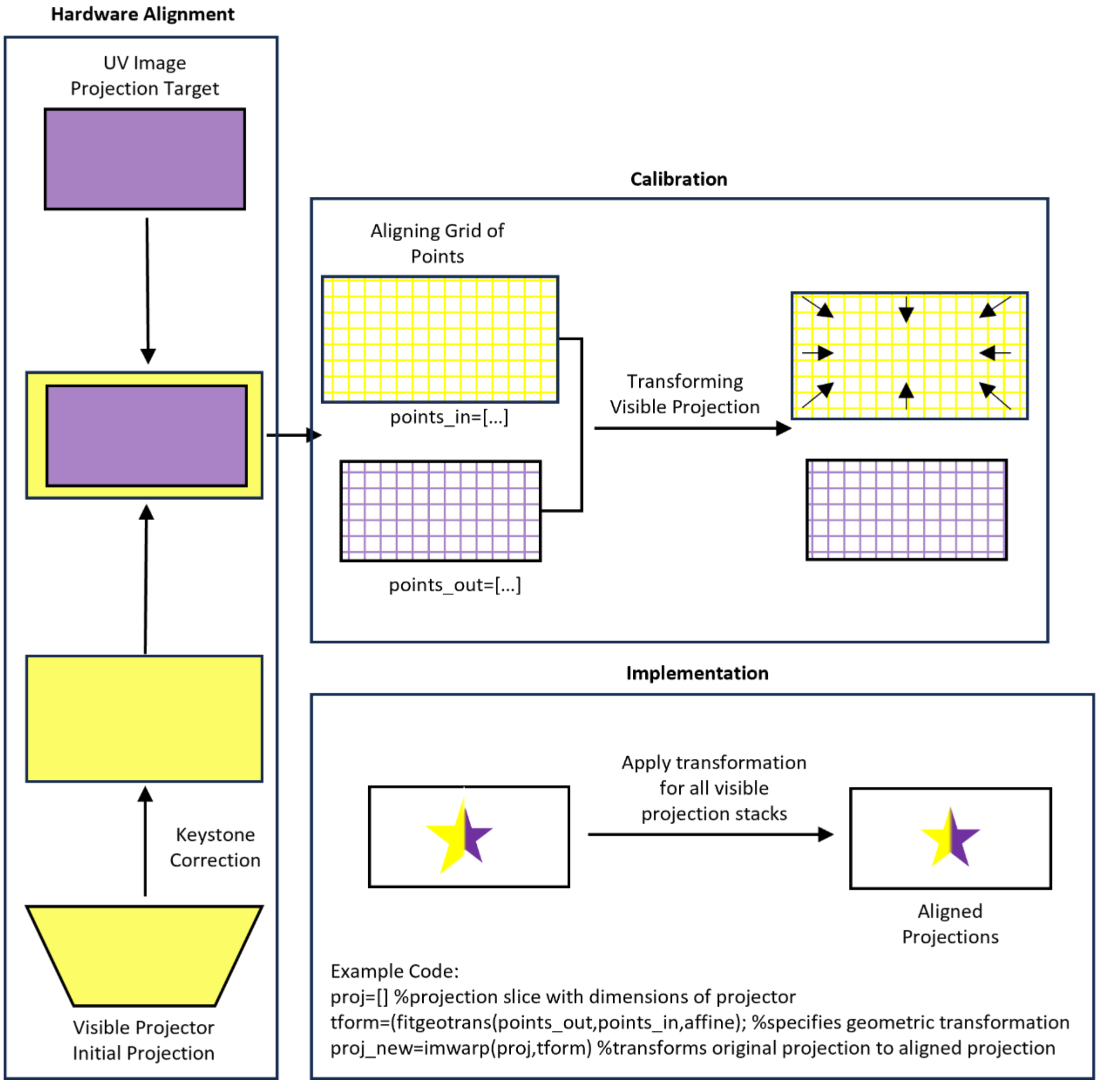

Supplementary Fig. 18: Flowchart showing the process used to align the visible and UV projector images. Both hardware and software were used to align images. The hardware alignment involved keystone correction to account for image distortion from projector angle. Then image transformation of each layer slice of the visible image is performed using a calibration by mapping a grid for each projector area.

Calibration Step (only needs to be performed once for a given printer setup):

1. Both projectors show a grid of 493 evenly spaced points across the entire projection area
2. The pixel coordinates for the intersection of the grid lines were selected from top left to bottom right in sequential order.
3. The same was done for the UV projection grid.
4. Each point was saved in an array.
5. Point coordinated of visible projector were stored in points_in array, while corresponding points on UV projector were stored in points_out array.
6. output_dim (resolution of UV projector), points_in (coordinates of clicked points on Visible projected image), and points_out (coordinates of of clicked points on uv projected image) were exported as 493_points.mat file, which is recovered during implementation step.

Implementation (align the larger projection, in our case the visible projector, to the smaller projection, in our case the uv projector)

1. The imwarp function was used to shrink the visible projection down to the UV projection using the point mapping previously identified in the calibration step (493_points.mat).
2. Pad the resized image on all sides using padarray, replicating edge values to fill new pixels. Since the edge values typically correspond to black color, it practically means we are assigning a 'black' color to fill the newly added borders, integrating them seamlessly with the original content of the image.
3. Repeat for all of the projections for a given sliced 3D model.

Supplementary Table 2: Printing parameters for dual wavelength printer

Supplementary Table 2: Print parameters used for dual-wavelength printing of multicolor resins from a single resin vat.

Resin	Visible Layer Time (sec)	UV Layer Time (sec)	Layer Thickness (µm)	Lift/Retract Distance (mm)
F1	4	180+	100	5
F5	25	160	100	5

Supplementary Fig. 19: Tensile data for multicolor samples

A

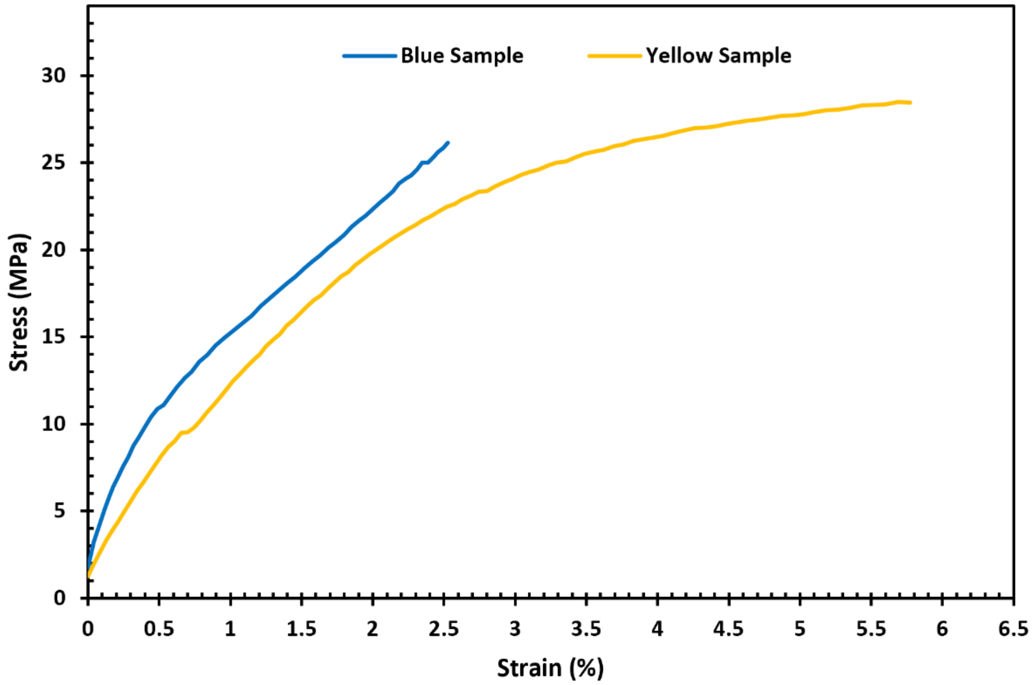

B

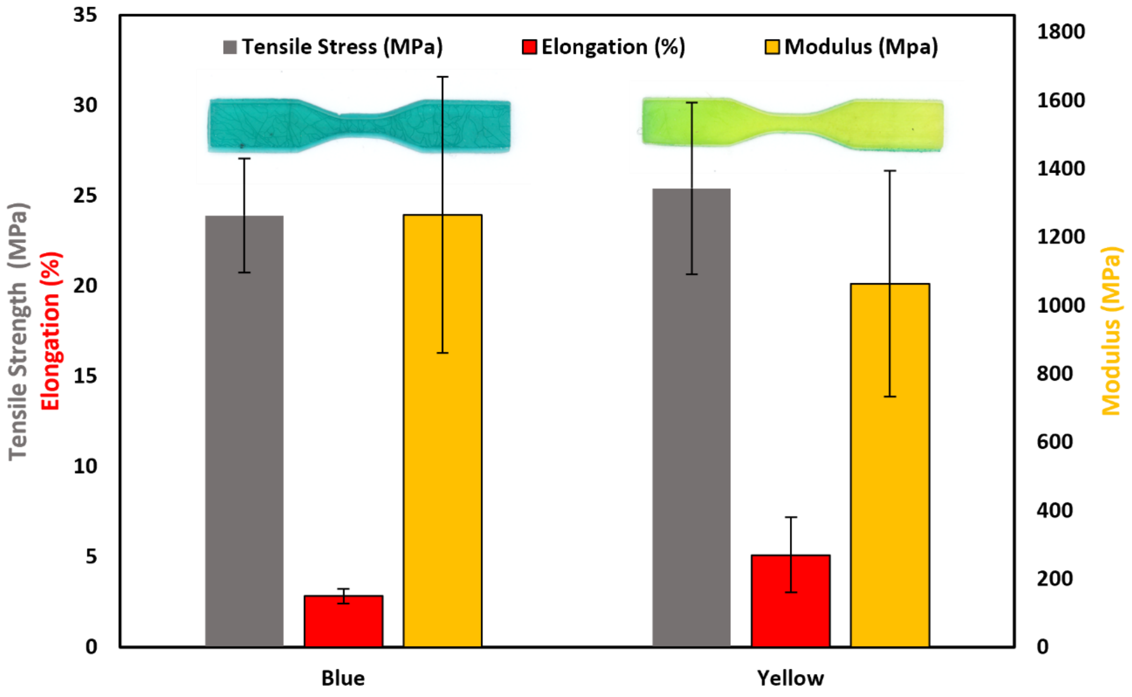

Supplementary Fig. 19: A) Representative tensile plot for blue and yellow samples under quasistatic tensile testing. B) Summary of tensile strength, elongation, and modulus results for quasistatic tensile testing with three replicates of blue and yellow samples. Error bars represent one standard deviation from three replicates.

Supplementary Fig. 20: Additional dual-wavelength 3D printed objects

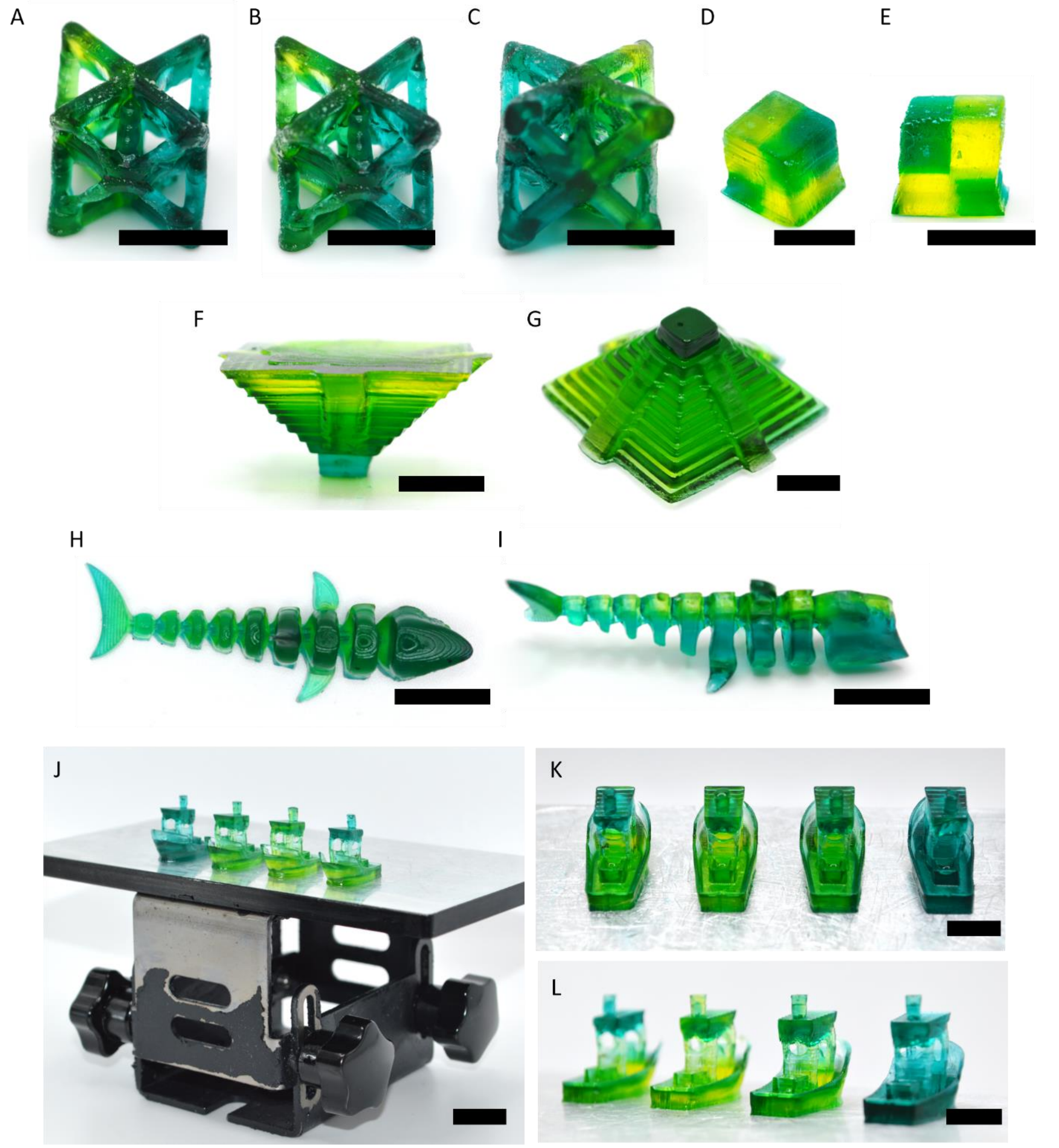

Supplementary Fig. 20: Additional images of dual-wavelength 3D printed objects. (A) Octet truss (B) Octet truss (C) Octet truss (D) Cube (E) Cube (F) Pyramid (G) Pyramid (H) Shark (I) Shark (J) Benchys (K) Benchys (L) Benchys. Scale bars=10 mm

Supplementary Fig. 21: White Lamp Light Source Emission Spectra

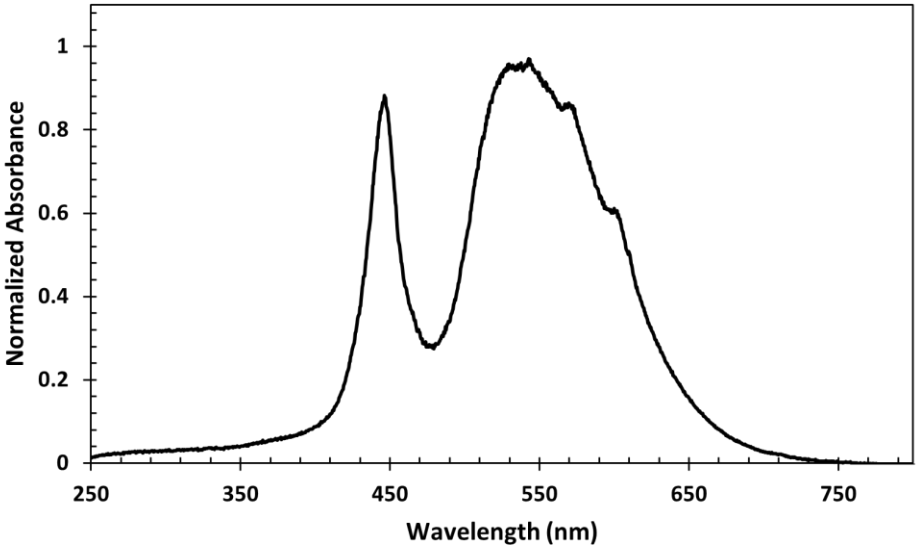

Supplementary Fig. 21: Normalized emission spectra for the white light lamp used to cure objects in PTFE molds.

Supplementary Fig. 22: 365 nm Kessil Lamp Light Source Emission Spectra

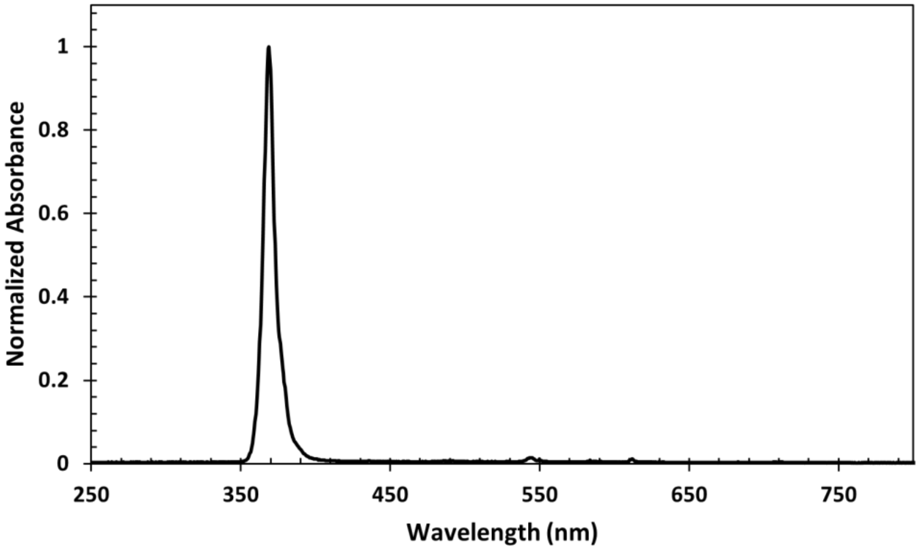

Supplementary Fig. 22: Normalized emission spectra for 365 nm Kessil lamp. The wavelength with max absorption was found to be 369 nm.

Supplementary Fig. 23: 456 nm Kessil Lamp Light Source Emission Spectra

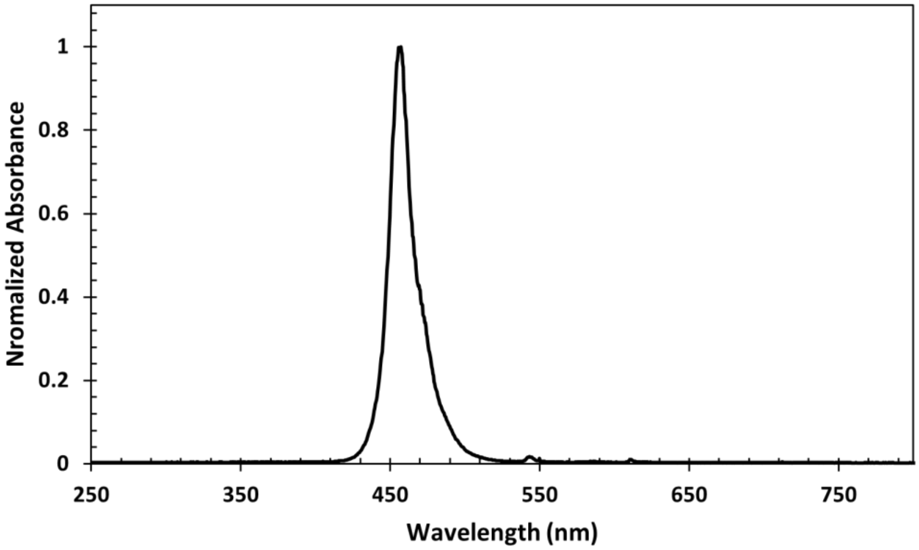

Supplementary Fig. 23: Normalized emission spectra for 456 nm Kessil lamp. The wavelength with max absorption was found to be 457 nm.

Supplementary Fig. 24: 405 nm Kessil Lamp Light Source Emission Spectra

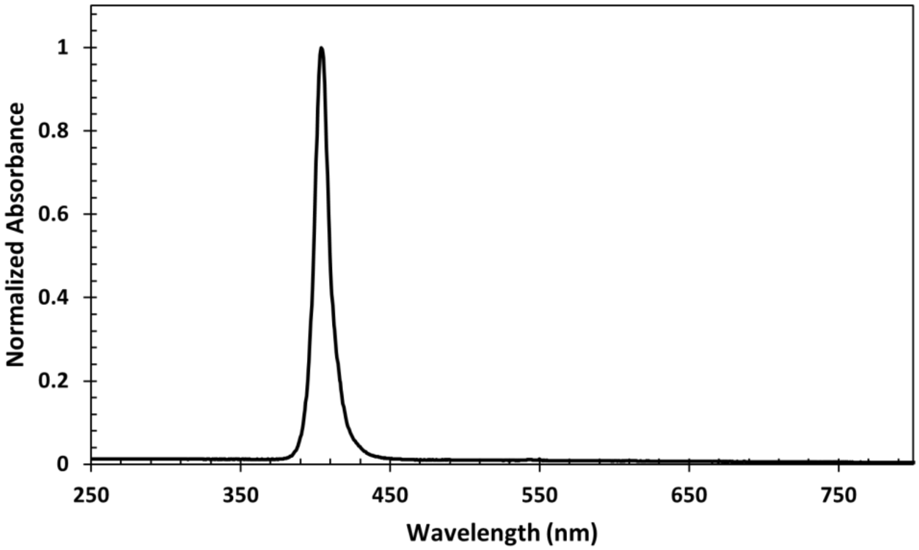

Supplementary Fig. 24: Normalized emission spectra for 405 nm light source. The wavelength with max absorption was found to be 404 nm.

Supplementary Fig. 25: 365 nm Kessil Lamp Light Source Emission Spectra

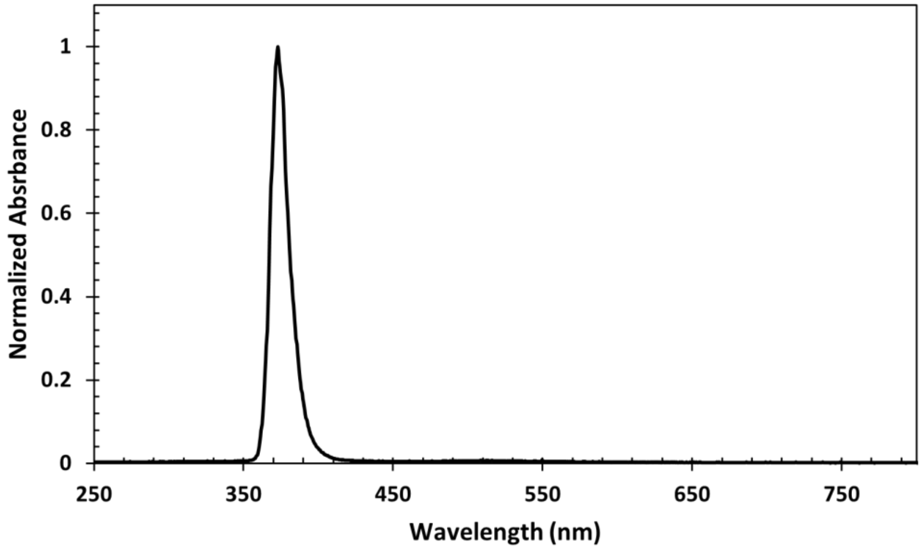

Supplementary Fig. 25: Normalized emission spectra for 365 nm projector. The wavelength with max absorption was found to be 373 nm.

Supplementary Fig. 26: White Light Projector Emission Spectra

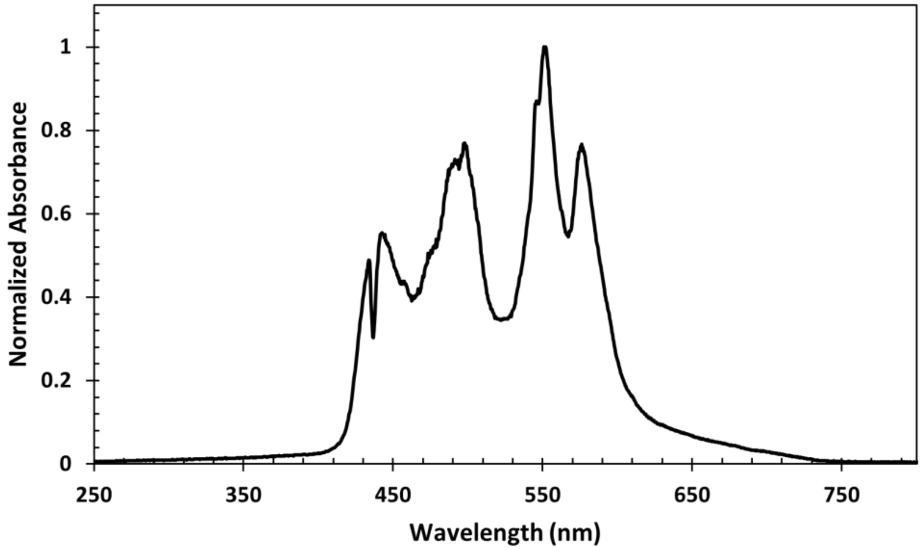

Supplementary Fig. 26: Normalized emission spectra for the white light projector.

Supplementary Fig. 27: Elegoo Mars 3 LCD 3D Printer Emission Spectra

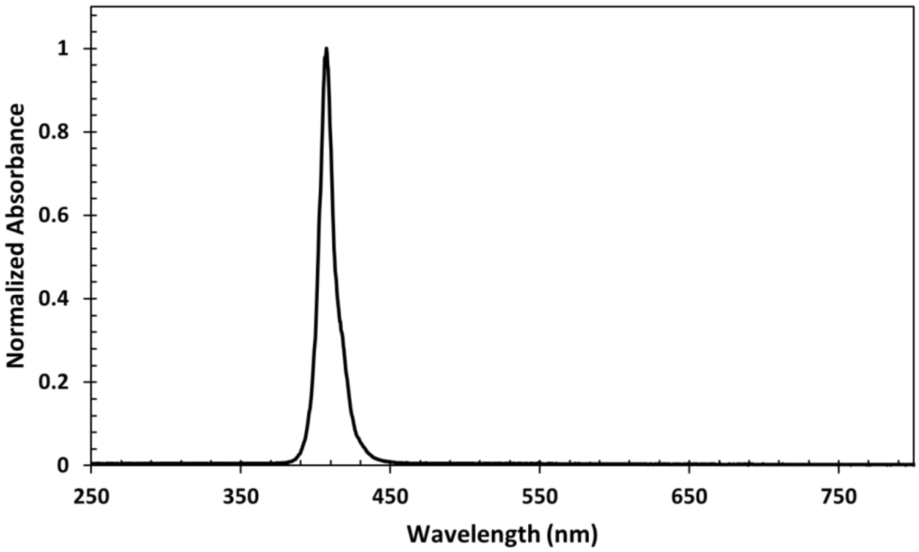

Supplementary Fig. 27: Normalized emission spectra for Elegoo Mars 3 LCD 3D printer.

Design Files

Shark: https://www.thingiverse.com/thing:3845304/files
Benchy: https://www.thingiverse.com/thing:5293974
Octet Truss: https://www.thingiverse.com/thing:5250099
Pyramid: https://www.thingiverse.com/thing:4155
