# Supplementary material for: Multi-color dual wavelength vat photopolymerization 3D printing via spatially controlled acidity
Source: Nat Commun. 2024 May 8;15:3867. doi: 10.1038/s41467-024-48159-7 (PMC11078982; doi:10.1038/s41467-024-48159-7)
Supplement: Supplementary file 3 — Description of Additional Supplementary Files [file 41467_2024_48159_MOESM3_ESM.pdf]

Description of Additional Supplementary Files

Supplementary Movie 1: Colored CAD model of design files next to GIF of the projected layer slices used for dual wavelength 3D printing.
